# Pathway-based Approach Reveals Differential Sensitivity to E2F1 Inhibition in Glioblastoma

**DOI:** 10.1158/2767-9764.CRC-22-0003

**Published:** 2022-09-23

**Authors:** Alvaro G. Alvarado, Kaleab Tessema, Sree Deepthi Muthukrishnan, Mackenzie Sober, Riki Kawaguchi, Dan R. Laks, Aparna Bhaduri, Vivek Swarup, David A. Nathanson, Daniel H. Geschwind, Steven A. Goldman, Harley I. Kornblum

**Affiliations:** 1 Department of Psychiatry and Biobehavioral Sciences, and Semel Institute for Neuroscience & Human Behavior, David Geffen School of Medicine, UCLA, Los Angeles, California.; 2 Voyager Therapeutics, Cambridge, Massachusetts.; 3 Department of Biological Chemistry, David Geffen School of Medicine, UCLA, Los Angeles, California.; 4 Department of Neurobiology and Behavior, School of Biological Sciences, UCI, Irvine, California.; 5 Department of Molecular and Medical Pharmacology, David Geffen School of Medicine, UCLA, Los Angeles, California.; 6 Department of Neurology, David Geffen School of Medicine, UCLA, Los Angeles, California.; 7 Department of Neurology and the Center for Translational Neuromedicine, University of Rochester Medical Center, Rochester, New York.; 8 The University of Copenhagen, Copenhagen, Denmark.; 9 Jonsson Comprehensive Cancer Center, UCLA, Los Angeles, California.; 10 Eli and Edythe Broad Center of Regenerative Medicine and Stem Cell Research, UCLA, Los Angeles, California.

## Abstract

Analysis of tumor gene expression is an important approach for the classification and identification of therapeutic vulnerabilities. However, targeting glioblastoma (GBM) based on molecular subtyping has not yet translated into successful therapies. Here, we present an integrative approach based on molecular pathways to expose new potentially actionable targets. We used gene set enrichment analysis to conduct an unsupervised clustering analysis to condense the gene expression data from bulk patient samples and patient-derived gliomasphere lines into new gene signatures. We identified key targets that are predicted to be differentially activated between tumors and were functionally validated in a library of gliomasphere cultures. Resultant cluster-specific gene signatures associated not only with hallmarks of cell cycle and stemness gene expression, but also with cell type–specific markers and different cellular states of GBM. Several upstream regulators, such as PIK3R1 and EBF1 were differentially enriched in cells bearing stem cell like signatures and bear further investigation. We identified the transcription factor E2F1 as a key regulator of tumor cell proliferation and self-renewal in only a subset of gliomasphere cultures predicted to be E2F1 signaling dependent. Our *in vivo* work also validated the functional significance of E2F1 in tumor formation capacity in the predicted samples. E2F1 inhibition also differentially sensitized E2F1-dependent gliomasphere cultures to radiation treatment. Our findings indicate that this novel approach exploring cancer pathways highlights key therapeutic vulnerabilities for targeting GBM.

**Significance:**

Molecular classification of GBM has not yet resulted in the development of effective therapies. We have developed an integrative approach to identify molecular targets differentially utilized by individual tumors. This approach could lead to patient- and tumor-specific therapeutics.

## Introduction

Glioblastoma (GBM) is incurable, with an overall median survival of approximately 14 months (1) despite standard-of-care treatment (2). The past decade has seen a revolution in the understanding of GBM, and studies of patient samples based on gene expression and oncogenic mutations have revealed that GBM can be parsed into distinct molecular categories—namely classical, mesenchymal, and proneural—and subsequently IDH-mutated tumors (3–8). While these classification schemes have shown some relationship to prognosis, they have largely failed to provide new therapeutic approaches.

The driver mutations of GBM result in the activation of many well-known oncogenic pathways (9). However, the use of pathway-specific inhibitors has not yet resulted in effective therapies. One potential explanation for this lack of efficacy is that tumors are comprised of multiple cell types with different pathway dependencies. Another is that inhibition of one pathway results in compensatory activation of other pathways (10). It is also likely that the identification of critical pathways and molecular targets driving GBM progression and recurrence is not yet complete. The prioritization of key pathways falls short of what would be required for tumor eradication because the combinatorial outcome of existing mutations, and resultant dominant pathways, cannot be conclusively inferred. Finally, novel therapeutic approaches will need to be put into the context of recent findings of intratumoral heterogeneity spurred by advances in single-cell technology. For example, we now appreciate that molecular subtypes previously used in the classification of GBM (5) can be linked to cell type–specific markers with the description of cellular states (11). Similarly, a subpopulation of tumor cells can express markers of outer radial glia and turn on developmental programs to promote invasion (12). Although these findings have advanced the field and opened new avenues of investigation, molecular and pathway vulnerabilities are still unknown in the different cell populations.

Here, we have developed a comprehensive approach to group samples based on their pathway utilization to uncover therapeutic targets. We hypothesized that, rather than examining GBM for individually expressed genes, analyzing targetable pathways in an unbiased manner could allow for the development of patient-specific or tumor class–specific therapeutics and combination therapies that go beyond traditional inhibitors. We have developed a bioinformatics strategy that leverages gene set enrichment analysis (GSEA) to disambiguate intertumoral heterogeneity in GBM**.** Using bulk RNA samples databases, we found that gene expression can be synthesized into gene signatures based on their enrichment for gene sets in the canonical and oncogenic collections. Furthermore, resultant gene signatures can be translated into potentially actionable molecular targets that signify functional and predictable differences between GBM tumors that can be used to identify potential therapeutic vulnerabilities in different cellular compartments. Finally, we utilized gliomasphere (GS) cultures and dependence on the pro-proliferative transcription factor E2F1 as an example to demonstrate the functional significance of our approach.

## Materials and Methods

### Patient and Tumor Datasets

GBM samples analyzed were composed of The Cancer Genome Atlas (TCGA) dataset (5), GS microarray dataset (13), and single-cell RNA sequencing (scRNA-seq) dataset (ref. 14; GSE57872).

### Patient-derived GS Cultures

Established patient-derived GS lines were cultured and maintained as described previously (15). Experiments were performed only with lines that were cultured for less than 20 passages since their initial establishment and short tandem repeat authentication was performed after passage 4 for each line. Cell lines were tested on initial culturing and every 2–3 months for *Mycoplasma* using the MycoAlert PLUS Detection Kit (Lonza).

### *In Vivo* Tumor Xenografts and Imaging

For tumor formation assessment, 8 to 12 weeks old NOD-SCID null (NSG, JAXID: 005557) mice were used in equal numbers of female and male and randomly assigned to experimental groups. A total of 5 × 10^4^ tumor cells infected with a firefly-luciferase-GFP lentiviral construct and either a scrambled or E2F1 short hairpin RNA (shRNA) vector were transplanted per mouse (*n* = 5), in accordance with University of California, Los Angeles–approved Institutional Animal Care and Use Committee protocols. Five mice were housed per cage, with a 12-hour light/dark cycle, and were provided food and water *ad libitum*. Tumor growth was monitored every 2 weeks after transplantation by measuring luciferase activity using IVIS Lumina II bioluminescence imaging. Regions of interest were selected to include the tumor area, and radiance was used as a measure of tumor burden. Mice were monitored and sacrificed upon the development of neurologic symptoms such as lethargy, ataxia, and seizures, along with weight loss and reduction in grip strength. Animals were sacrificed by CO_2_ asphyxiation and secondary cervical dislocation.

### GSEA, Gene List Generation, and Target Prediction

For TCGA and GS datasets, each sample was compared with the average of the whole dataset using the canonical (C2CP) and oncogenic (C6) gene set collections from the GSEA website (http://www.gsea-msigdb.org, SCR_003199). Enrichment profiles were then used to generate principal component analysis (PCA) plots, and the contribution of each gene set to a particular direction was extracted using R-package “FactorMineR.” The top 20 contributing gene sets in a particular direction were compared with one another and common elements (present in at least five gene sets) were employed in each gene signature. The signature names are an acronym derived from each dataset (TCGA or GS), the GSEA collection (2 = canonical, 6 = oncogenic), a dash followed by the component number (PC1 or PC2), and direction of the correlation (positive or negative). For example, T2-1N represents TCGA, canonical, PC1, negative correlation (see Supplementary Table S1). Datasets were clustered with these gene signatures for downstream analyses. Gene signatures were interrogated via Ingenuity Pathway Analysis (IPA; ref. 16; SCR_008653) using the upstream regulator tool to predict targets (molecules or drugs) as either activated or inhibited.

### Single-cell Signature Score Analysis

Single-cell expression data (*n* = 430) from five primary human GBM specimens were imported from GSE57872. For each gene set of interest, single-cell enrichment scores were generated as described previously (14). Briefly, the enrichment score of a gene set was computed in each cell by taking the average expression of genes within the gene set and subtracting the average expression of all detected genes. Single-cell enrichment scores were generated for (i) the six TCGA/GS gene lists discussed above (ii), the cell-cycle meta-signature described in ref. 14), and (iii) the stemness signature described in ref. 17. These scores were used to visualize pairwise gene set correlations across cells, specifically between each of the six TCGA/GS gene lists and cell cycle or stemness. Single-cell enrichment scores were then generated for two additional groups of gene sets: developing (18) and adult (19) brain cell type markers and GBM cellular state markers (11). Correlation plots were generated using the “corrplot” package in R. Displayed are the correlation coefficients for each pair and circles whose color and size reflect the coefficient value and magnitude, respectively. For pairs with nonsignificant correlation, the coefficients are displayed without circles. Significance was evaluated using α = 0.05 for both raw (below diagonal) and FDR-adjusted (above diagonal) *P* values. Correlation patterns were used to group the gene signatures through hierarchical clustering, with black boxes marking the resultant clusters.

### *In Vitro* Functional Analysis: Sphere Formation and Cell Proliferation

Cell proliferation experiments were conducted by plating cells at a density of 2,000 cells/well in a 96-well plate in quadruplicate. Cell number was measured after 3 and 7 days and normalized to the initial reading at day 0 using the CellTiter Glo Luminescent Cell Viability Assay (Promega). The experiments shown represent fold change at day 7 relative to day 0. For sphere formation assays, cells were plated at a low density (100, 50, 25, and 12 cells per well) in 96-well plates (24 wells per density). Cells were maintained for 10 days before sphere formation was evaluated. Spheres larger than 10 cells in diameter were considered for analysis. The numbers shown represent the number of cells per well or the stem cell frequency as calculated using the Walter and Eliza Hall Institute Bioinformatics Division ELDA analyzer (http://bioinf.wehi.edu.au/software/elda/; ref. 20). All sphere formation and proliferation experiments were repeated at least three times.

### Lentivirus Transduction in GS Lines

Lentiviral vector particles containing E2F1 and scrambled shRNAs were purchased from Abmgood (catalog no. 188270910496). Cells were transduced with the corresponding viruses for 48 hours and selected with puromycin (Sigma). Knockdown of E2F1 was confirmed using immunoblotting in treated samples. In addition, we also tested CRISPR constructs targeting E2F1 (Abmgood, 188271110595) using the same lentivirus technology.

### Immunofluorescence Analysis

Cells were plated on 24-well plates pretreated with laminin overnight. After 2 days of culture, cells were fixed in 4% paraformaldehyde for 15 minutes at room temperature, followed by blocking and overnight incubation at 4°C with γH2AX primary antibody (Cell Signaling Technology, AB_10860771). Cells were then incubated with species-specific goat secondary antibody coupled to AlexaFluor dye (568, Invitrogen) and Hoechst dye for nuclear staining for 2 hours at room temperature. Plates were imaged using EVOS microscope, and quantification of positively stained cells was performed manually using ImageJ (SCR_003070).

### Irradiation of GSs

Cells were irradiated at room temperature using X-ray irradiator (Gulmay Medical Inc.) at a dose rate of 5.519 Gy/minute for the time required to apply an 8 Gy dose. The X-ray beam was operated at 300 kV and hardened using a 4 mm Be, a 3 mm Al, and a 1.5 mm Cu filter, and calibrated using NIST-traceable dosimetry.

### Statistical Analysis

Reported data are mean values ± SEM for experiments conducted at least three times. Unless stated otherwise, one-way ANOVA was used to calculate statistical significance, with *P* values detailed in the text and figure legends. *P* values less than 0.05 were considered significant. Correlation analyses were performed using Pearson coefficient. Log-rank analysis was used to determine the statistical significance of Kaplan–Meier survival curves. Data analysis was done using R v 3.6.3 (21).

### Data Availability Statement

The data analyzed in this study were obtained from Gene Expression Omnibus at GSE57872. The data generated in this study are available within the article and its Supplementary Data.

## Results

### GBM Clustering is Clinically Significant and Only Modestly Overlaps with Prior Molecular Classification

**FIGURE 1:**
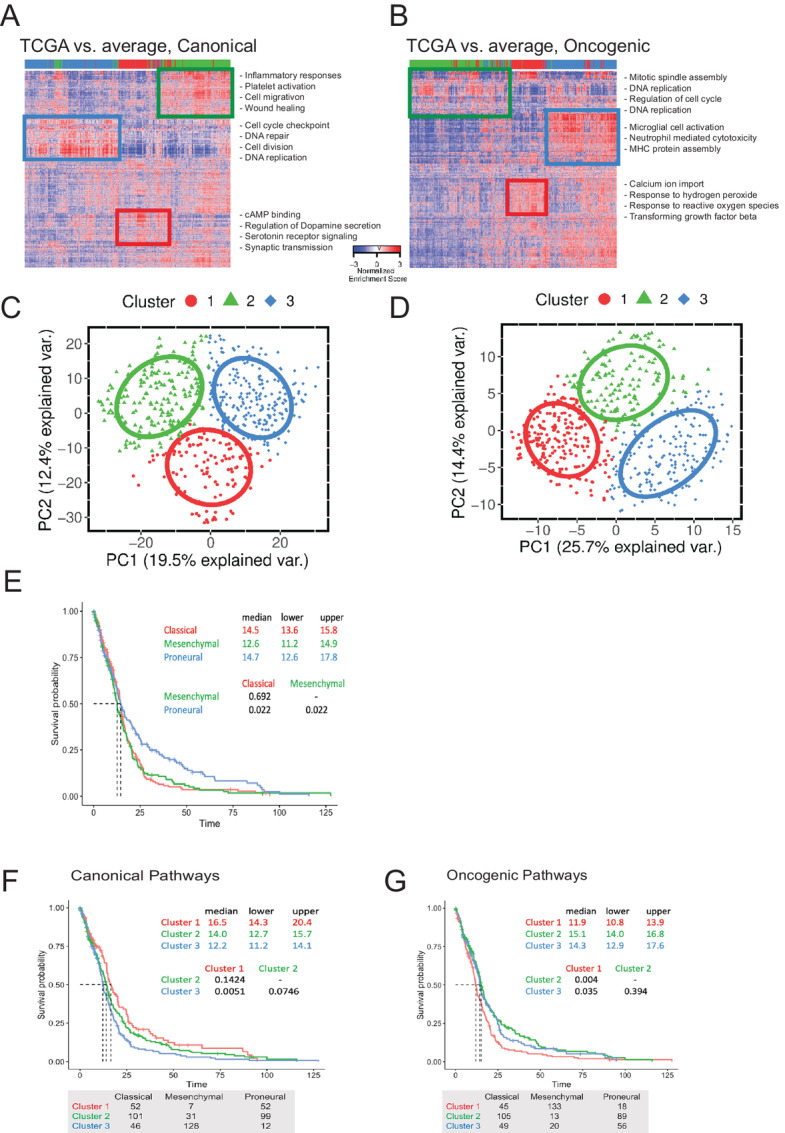
Pathway-based analysis generates three distinct clusters based on enrichment profiles with clinical significance. Samples from TCGA were analyzed using either canonical pathways (**A**) or oncogenic pathways (**B**) from the GSEA to generate heatmaps based on the enrichment profile of each sample (column) with respect to each gene set (row) in both collections. **C** and **D,** Profiles from **A** and **B**, respectively, were used to generate PCA plots labeled by color and shape for each cluster. Circle lines represent the normal distribution of the samples in each cluster. **E,** TCGA samples were clustered on the basis of the original molecular subtypes described, and Kaplan–Meier curves were obtained. **F** and **G**, Samples clustered on the basis of enrichment profiles for canonical and oncogenic gene sets, respectively, were analyzed for survival using Kaplan–Meier curves. Tables at the bottom describe the distribution of the molecular subtypes for each cluster. Dotted lines represent median survival for each curve (also described in top tables). Time shown is in months. *P* values after *post hoc* analyses using Bonferroni–Hochberg correction.

To characterize heterogenous pathways activated in GBM, we analyzed GBM patient samples in TCGA (5) using gene sets in the canonical (C2CP) and oncogenic (C6) pathway collections from GSEA (22, 23). These gene sets were selected to analyze known pathways (canonical) and those that have been described as prevalent in cancer that could be targeted pharmacologically (oncogenic). The mRNA expression of each TCGA sample was compared with the average expression of all the samples (*n* = 538), and normalized enrichment scores were obtained for all the gene sets comprising the two pathway collections. We observed heterogeneity when either canonical (Fig. 1A) or oncogenic (Fig. 1B) gene sets were analyzed. We determined that three clusters represented the data when using oncogenic and canonical pathways via non-matrix factorization and consensus clustering; the robustness of the clusters was also tested and validated using the random forest approach (Supplementary Figs. S1A–S1F). We then applied PCA for dimensionality reduction and to better visualize the sample clusters (Fig. 1C and D, respectively). Notably, when the clustered samples were colored by their known TCGA molecular subtype, we found there was one cluster that contained approximately 75% of the mesenchymal samples, while the classical and proneural subtypes were present in all clusters (Supplementary Fig. S1G). Because of the lack of precise correspondence between TCGA subtype and our pathway-based classification, we hypothesize that TCGA classification may not represent true functional differences between tumors and that a pathway-based approach would reveal heterogeneity that would be more susceptible to therapeutic intervention as described below.

To determine whether our pathway-based classification was of prognostic value, we examined patient survival using the pathway-based clustering methodology. Prior studies using TCGA groupings have found only limited association with survival, with proneural tumors having longer survival—an observation largely driven by the subset of IDH-mutant tumors. As shown in Fig. 1E, our own analysis of TCGA categories found significant differences only between the proneural and other two groups, as reported previously (5). However, when we utilized our new pathway-based clustering approach, we found statistically significant differences in median survival between the patients from clusters 1 compared with 3 (canonical) and cluster 1 compared with cluster 2 and 3 (oncogenic; Fig. 1F and G). For both collections of gene sets, canonical and oncogenic, the cluster with the lowest median survival was the one primarily composed of mesenchymal samples. However, approximately 30% of the samples within this cluster were characterized as classical and proneural. Similarly, using the canonical pathways collection, the cluster with the highest median survival included equal abundances of classical and proneural samples (47% each). These data challenge the idea that samples obtained from patients should be treated according to their TCGA-defined molecular phenotype and, in contrast, support the notion that tumors from different molecular backgrounds might have common signaling pathways.

### GSEA Gene Signatures From a Patient-derived GS Database Delineate Potentially Actionable Targets

**FIGURE 2:**
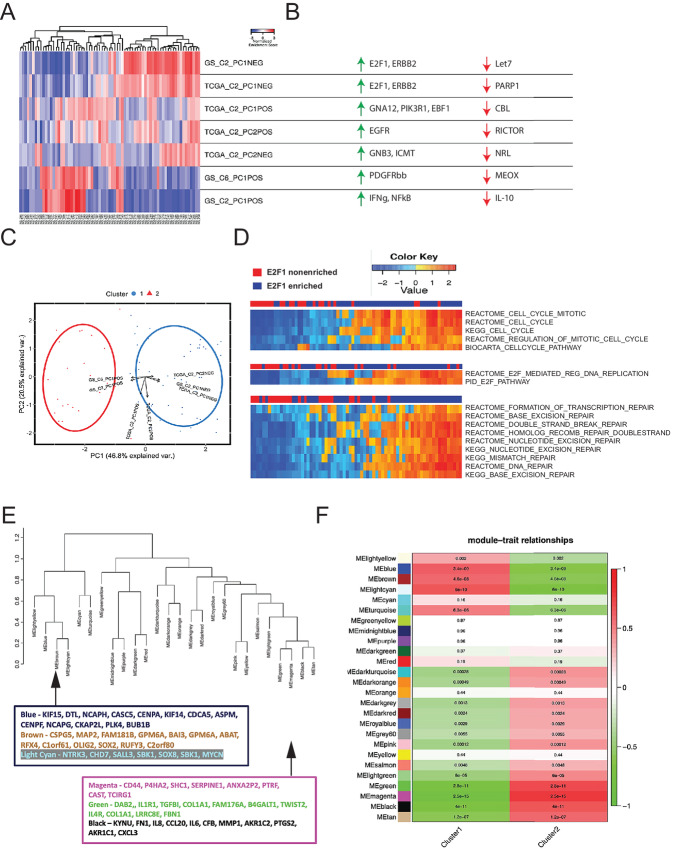
Gene lists predict E2F1 as a main target in one of the clusters found in the GS dataset. **A,** Enrichment profiles using gene lists were generated for GS samples. **B,** Each gene list was evaluated using IPA and top predicted activated (green arrows) and inhibited (red arrows) upstream regulators are shown. **C,** PCA plot from enrichment scores generated in **A** showing how each gene list contributes to a particular direction. **D,** Samples from both clusters were evaluated for their enrichment of cell cycle–related, downstream E2F1 target, and DNA damage repair gene sets from the canonical pathway collection. **E,** WGCNA generated 26 modules when samples were analyzed on the basis of their enrichment profiles for the gene lists. **F,** Modules are ranked on the basis of their abundance in both clusters. Modules at the top are highly enriched in the E2F1-activated cluster (cluster 1).

We recognized that GSEA is an imperfect approach to assess functional pathway utilization and that individual genes or sets of genes would contribute to enrichment of multiple gene sets. Therefore, to more pragmatically develop potential interventions based on our analysis, we further distilled our pathway-based clustering of the whole TCGA dataset by extracting the top contributing gene sets to each principal component (PC) and direction and synthesizing their common elements into gene signatures. We hypothesized that targeting the most highly represented elements would allow us to engage several key pathways simultaneously, even though we might be limiting our scope to shared targets and ignoring underrepresented pathways. To validate this approach, we performed a similar analysis on a microarray-derived database of patient-derived GS lines so that we could functionally test downstream targets (13). The enrichment patterns again showed heterogeneity between samples, yet they grouped into two major clusters when either canonical and oncogenic gene set collections were used (Supplementary Figs. S2A and S2B). Gene signatures were also generated for the GS dataset based on the common elements shared among the top contributing gene sets for each PC and direction, as described above for TCGA dataset (Supplementary Table S1). The TCGA- and GS-based gene lists were then used to obtain enrichment scores in the GS lines (Fig. 2A). This dataset again separated into two main clusters in accordance with the pattern of enrichment scores generated when the oncogenic and canonical pathways were used (Supplementary Fig. S2C). To convert gene signatures into actionable targets, we further analyzed them through IPA. Cluster 1 (right side of heatmap in Fig. 2A) showed higher enrichment of the G2-1N gene signature and core analysis from IPA showed an increase in the expression of the E2F family of transcription factors and its downstream targets (Fig. 2B). The contribution of each gene signature can be appreciated in reference to both clusters (Fig. 2C). Activation of E2F1, together with inhibition of Let7, which has been reported to have a role in differentiation and tumor suppression (24, 25), had the most significant *P* value for this gene signature. Because E2F1 has a known role in cell-cycle progression, we examined the enrichment scores for cell cycle–related and E2F1-target gene sets in the canonical pathways collection (C2CP). Indeed, samples that fell in the cluster with high enrichment of G2-1N (and hence predicted to have activation of *E2F1*) had concomitant higher enrichment scores for cell cycle and *E2F1* activation (Fig. 2D). E2F1 has also been shown to be induced in response to radiation and chemotherapy (26, 27). Analysis of DNA damage response gene sets also revealed an enrichment in the samples predicted to have *E2F1* activation (Fig. 2D). These findings suggest that there are two clusters of GS samples based on their enrichment of specific gene signatures that can be further analyzed to elucidate upstream regulators. Among the most meaningful differences between the samples was the fact that one cluster revealed an *E2F1*-activated expression exhibiting a high degree of enrichment for cell cycle and DNA damage response signaling expression. Moreover, the two clusters had clinical significance with the *E2F1*-activated group showing better median survival compared with the other cluster (Supplementary Fig. S2D). As was the case with TCGA dataset, stratifying patients using molecular subtypes did not show differences in survival (Supplementary Fig. S2E).

We next reclustered TCGA samples based on their enrichment for the gene signatures described above and found three clusters, comparable with the original pathway analyses described (Supplementary Fig. S3A). The *E2F1*-activated signatures (T2-1N and G2-1N) characterized one of the clusters, whereas the *EGFR* signature (T2-2P and G2-2P) pointed in between two of the clusters. In addition, we analyzed raw data from the available TCGA samples (*n* = 160) from Broad Firehose. Differential expression analysis was performed on samples using the cluster identity from the gene signatures. These data were then used to highlight the most enriched gene ontology terms for each cluster. We found each of the three clusters had a defined set of Gene Ontology terms: cell cycle–related (cluster 2), extracellular membrane and inflammation (cluster 3), or synapse and neurotransmitter signaling (cluster 1; Supplementary Fig. S3B). These results suggest the existence of distinct clusters that can be parsed through their potential pathway utilization, highlighted by upstream regulator enrichment. Similarly, we found a greater complexity in TCGA dataset compared with the GS dataset, as would be expected when analyzing primary and patient-derived lines, respectively, as patient-derived lines do not encompass nontumor cells present in the original TCGA samples and likely represent a less complex mixture of cells than found in the tumor.

### Weighted Gene Coexpression Network Analysis Reveals Distinct Regulatory Modules in Each Cluster

To further determine possible coregulated subsets of genes, we performed weighted gene coexpression analysis (WGCNA) on differentially expressed genes. This analysis has been used to identify gene networks with central hub genes that are of critical functional importance (28). We identified six modules correlated with cluster 1 and 20 modules correlated with cluster 2 (Fig. 2E and F). We took the top three enriched modules for each cluster and performed gene ontology enrichment analysis. The top modules associated with E2F1 activation (blue, brown, and light cyan) showed enrichment of cell cycle, cell division, and DNA replication, in addition to processes associated with neurogenesis, neuron differentiation, and gliogenesis (Supplementary Table S2). Moreover, two of the three modules also showed enrichment in their promoter region for *E2F1* and other members of the E2F family.

In contrast to the E2F1 module, the top modules associated with the non–E2F1-enriched cluster (black, green, and magenta modules) showed enrichment for inflammatory response, cell migration, and chemotaxis, as well as immune response, angiogenesis, and regulation of apoptotic processes. In these E2F1-independent modules, we found genes with enrichment for transcription factors involved in inflammatory responses, such as CEBPB and the interferon regulatory transcription factor family, as well as C2H2 zinc finger family members, including EGR1 and SP/KLF, which regulate proliferation, differentiation, and apoptosis cellular processes. We then identified hub genes in each module, determined by how associated they are to the other members of their module (28), and colored them by module name in Fig. 2E. Like the modules, the hub genes had different characteristics for modules associated with the *E2F1* activated as compared with the *E2F1*-independent clusters. Namely, the enriched *E2F1*-related modules include hub genes of known stem cell markers *SOX2* and *OLIG2*, associated with self-renewal and persistent proliferation, as well as markers of cell division, like PLK4. The hub genes of the *E2F1* independent–related modules include *IL8*, *IL6*, other inflammatory cytokines, and *CD44*, which has been associated with a more invasive phenotype.

From a clinical perspective, we wanted to know whether hub genes would be viable as therapeutic targets. To this end, we examined the data from a prior study comparing gene expression in the cellular fraction containing tumor initiating cells, termed glioma-derived progenitors cells (GPC) and normal, nontransformed glial progenitor cells (nGPC; ref. 29). For the E2F1-related modules, several hub genes (*DTL, CASC5, CDCA5, ASPM, CENPF, BUB1B*; blue module) had expression at least 4-fold higher in GPCs as compared with nGPCs. Similarly, another hub gene in an *E2F1*-related module, *MYC* (light cyan module) was 12-fold more highly expressed in GPCs relative to nGPCs. Evaluation of the *E2F1*-independent associated hub genes uncovered that *CD44* and *COL1A1*, both associated with invasion, were highly expressed in GPCS (over 22-fold change higher related to nGPCs). Two other genes associated with migration and invasion, *FN1* and *SERPINE1*, were also at least 4-fold higher in GPCs in the E2F1-independent cultures. This analysis indicates that targeting the modules and hub genes identified for each cluster would likely have fewer off target effects based on their expression restricted to GPC as opposed to normal progenitor cells.

### Gene Signatures Differentially Associate with Cell Cycle and Stemness Programs, GBM Cellular States, and Cell Type–specific Programs

One main focus of GBM research in the last decade has been the existence of a subset of cancer cells with activated stemness programs, namely glioma stem cells, that contribute to the malignancy of the tumor (30–33) and are refractory to therapy (34, 35). Together with TCGA molecular subtypes, this paradigm has resulted in the development of specific therapies aimed at targeting molecules believed to be key regulators of tumor growth and invasion. As we routinely work with patient-derived cell lines that behave differently than tumor cells in their intact tumor microenvironment in patients, we need to establish whether critical pathways such as stem-like programs, differentiation pathways, or cell cycle–related signatures are predominantly active in these cells. To that end, we evaluated the association of our pathway-based gene signatures with cell-cycle and stemness scores in the single-cell RNA-seq dataset (14). Consistent with our GS analyses, we found that both of our signatures predicted to have *E2F1* activation (G2-1N and T2-1N) strongly correlated with cell-cycle scores (Supplementary Fig. S4A), while other signatures showed weak or no association. Interestingly, we found two signatures (T2-1N and T2-2P) that had strong negative correlations with stemness score and one with a positive correlation (T2-1P; Supplementary Fig. S4B).

The negative correlation between activation of *E2F1* (T2-1N) and stemness score is not surprising given the fact that tumor cells are believed to be in either a proliferative or a stemness state (34). However, *EGFR* activation (T2-2P) has not been linked to a decrease in stemness and compels further investigation. The gene list with positive correlation to stemness (T2-1P) has three main targets per IPA analysis. First, phosphatidylinositol 3-kinase (PIK3R1) has been associated with GBM malignancy, and there are several inhibitors developed for molecules in this signaling pathway (36). GNA12 encodes for the G12 alpha subunit of G proteins and is of critical importance in regulating actin cytoskeletal remodeling in cells during migration, which is critical for tumor invasion. Finally, early B-cell factor 1 (EBF1) has been identified as a TET2 interaction partner in IDH-mutant cancers (37). These analyses establish a novel approach for uncovering new molecular targets based on a pathway-based approach that can be leveraged for the development of new therapies.

**FIGURE 3:**
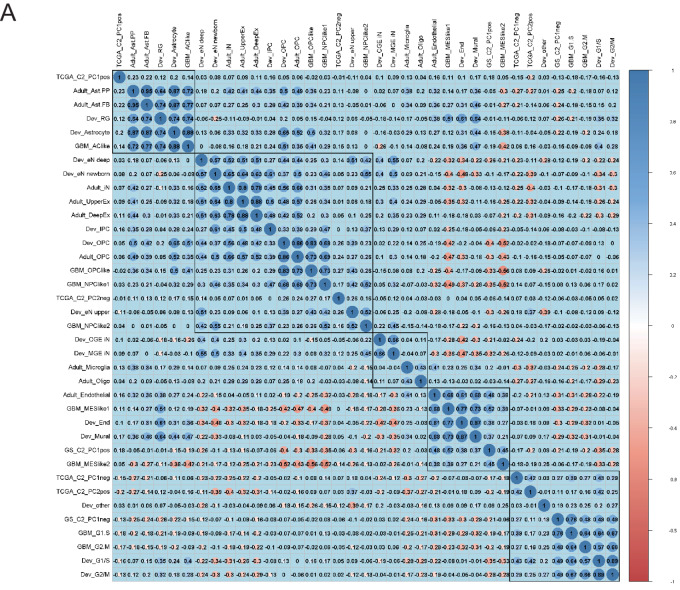
Gene lists differentially correlate with cellular states and cell-specific markers. **A,** Scores generated for each cell in Supplementary Fig. S3 using gene lists were correlated with scores for cellular states and specific cell-type markers in development and adult brain. The presence of a circle represents significant correlation, and the size and color depict the intensity of the correlation. Boxes mark groups of gene lists strongly correlated (based on hierarchical clustering).

Next, we sought to determine the relationships of our gene signatures to other published gene signatures. These associations will allow us to get a better understanding of upstream regulators targets and cellular states and identities. We used the same scRNA-seq dataset (14) and compared our gene signatures to previously reported transcriptional signatures of cell types in adult cortex (19), developing human brain (18), and to cellular states in GBM (11). As expected, our E2F1-activated gene lists both significantly correlated with G_1_–S and G_2_–M signatures (Fig. 3A). In addition, T2-1P (PIK3R1 and EBF1 activated) significantly associated with adult astrocytic markers and the AC-like molecular state, and T2-2N (ICMT activated) significantly associated with adult oligodendrocyte progenitor cell (OPC) markers and both OPC-like and neural progenitor cell (NPC1)-like molecular states. Notably, ICMT is a methyl transferase necessary for the localization of CaaX proteins (which include the Ras family) to the cell membrane (38, 39). Ras/ERK signaling has been associated with the proneural subtype (40) and NRAS is expressed at higher levels in proneural subtype compared with mesenchymal and classical (https://gliovis.bioinfo.cnio.es/). TCGA samples classified as proneural also showed higher enrichment for genes in the NPC-like and OPC-like molecular states (11). These data suggest a targetable mechanism (trafficking to the cell membrane via ICMT) required for a specific protein (Ras) upregulated in a TCGA subtype (proneural) that has correlates in GBM molecular states (OPC-like, NPC1-like). Finally, we also found a significant correlation between G2-1P (*IFNγ* and *NFκB* activated) with both MES1-like and MES2-like states as well as adult endothelial and mural cell markers. The latter include blood vessel–associated cell types such as pericytes and vascular smooth muscle cells. This association relates to the extraordinary plasticity of glioma cells in response to their microenvironment. These data suggest IFNγ and NFκB pathways are activated in cells in the mesenchymal states that undergo vascular mimicry and express markers related to endothelial cells and pericytes that have been associated with tumor progression and recurrence (41–43). Of note, *IL10* is also predicted to be inhibited in this gene list; given IL10 is an anti-inflammatory cytokine, this suggests an inflammatory microenvironment would promote this particular molecular state. All these associations are summarized in Supplementary Table S3.

### Differential Effects of E2F1 Silencing and Candidate Therapeutics Support the Functional Significance of Pathway-based Heterogeneity

**FIGURE 4:**
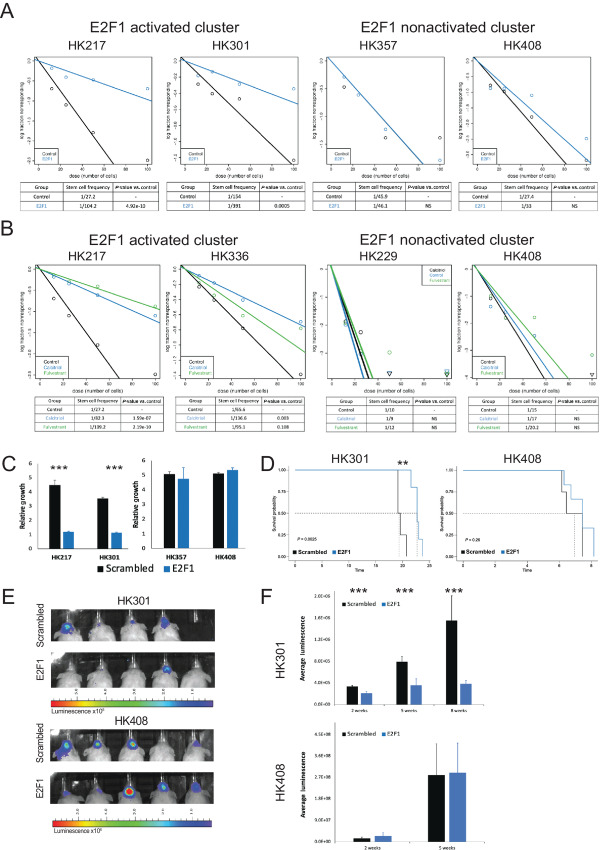
E2F1 silencing compromises self-renewal and proliferation *in vitro* and tumor formation *in vivo*. Samples from both clusters were treated with control (scrambled) or E2F1 siRNA (**A**) or were plated in regular media or media containing fulvestrant or calcitriol (**B**) under limiting dilution in a 96-well plate. Graphs depict the number of wells that did not form spheres after 10 days versus the number of cells plated (a vertical line implies all wells formed spheres). **C,** Cells treated with scrambled or E2F1 siRNA were plated at a density of 2,000 cells per well in a 96-well plate in quadruplicate, and their growth was evaluated using luminescence. Relative growth is the fold change compared with basal measurement. **D–F**, Cells treated with scrambled or E2F1 shRNA were intracranially injected in NSG mice. Kaplan–Meier survival curves for each group was calculated; dashed lines represent median survival and time shown is in weeks (**D**). Luminescence was assessed 2 weeks after transplantation (**E**). Quantification for each group is shown at 2, 5, and 8 weeks (**F**). Mice in both groups for HK408 did not reach the 8-week timepoint. Experiments in **A–C** were performed at least three times. Data are represented as mean ± SEM. **, *P* < 0.01 and ***, *P* < 0.001 as assessed by one-way ANOVA.

Our findings of potentially differential dependence on E2F1 and its downstream targets in two groups of GS cultures, both of which were actively proliferative was somewhat surprising, as this transcription factor is often thought to be primarily involved in proliferation and cell-cycle regulation. To investigate this further and to validate our general approach, we used silencing technology to evaluate the cellular effects of E2F1 suppression in samples from *E2F1*-activated and *E2F1*-independent clusters. HK217 and HK301, members of the E2F1-activated cluster, showed a marked decrease in stem (sphere-forming) cell frequency in a limiting dilution assay (LDA) in cells with E2F1 knockdown compared with control (Fig. 4A). These effects were not observed in HK357 or HK408, lines that were not enriched for an E2F1 signaling pathway signature (Fig. 4A), even though both cultures expressed E2F1. To confirm the effects of the knockdown, we used CRISPR/Cas9 to delete E2F1 in activated and nonactivated cultures and performed LDA, with a similar outcome: That genetic disruption of *E2F1* in activated cultures showed diminished sphere-forming capacity and had little effect in the non–*E2F1*-activated cells (Supplementary Fig. S5).

In addition to our genetic approach, we utilized a pharmaceutical approach to highlight the importance of the gene list identified. In addition to *E2F1* activation (Fig. 2B), IPA also predicted calcitriol and fulvestrant would have an inhibitory effect upon the genes enriched in the *E2F1*-activated cluster. We tested this hypothesis by performing LDA in cells from both E2F1-activated and -nonactivated clusters (Fig. 4B). Both *E2F1*-activated GS lines, HK217 and HK336, showed a decrease in sphere formation capacity when treated with calcitriol (10 nM) while fulvestrant (10 μM) showed a dramatic decrease in HK217 and a modest, yet not significant, inhibition in HK336 (Fig. 4B). Both GS lines in the nonactivated cluster, HK229 and HK408, did not show differences when treated with either drug compared with the control group. Likewise, knockdown of E2F1 resulted in compromised overall cell proliferation in *E2F1*-enriched samples when E2F1 expression was suppressed (Fig. 4C), compared with *E2F1*-independent cells where E2F1 knockdown did not significantly alter proliferation.

To validate our approach and our fundamental findings of differential sensitivity to E2F1 knockdown, we analyzed an independent RNA-seq dataset derived from 22 GSs and generated enrichment scores for the gene lists identified previously (Supplementary Table S4).

As we previously observed in our larger set of GSs, there was heterogeneity in the E2F1-related enrichment scores (Supplementary Fig. S6A). On the basis of the resulting scores, we treated GS024 (*E2F1* enriched) and GS102 (*E2F1* nonenriched) with control or E2F1 knockdown lentivirus and, as expected, we observed a significant decrease in sphere formation capacity after E2F1 knockdown in GS024 but not GS102 (Supplementary Fig. S6B). These data tested the predictive capacity of our approach in an independent dataset and showed we were able to target a predicted protein of interest with functional implications.

To determine the *in vivo* relevance of our findings, we assessed tumor formation capacity in HK301 (*E2F1*-dependent) and HK408 (*E2F1*-independent) cell cultures transduced with shE2F1 or shControl (scrambled) lentivirus mediated gene expression targeting. Kaplan–Meier survival curves showed an increase in median survival in cells treated with shE2F1 (22.7 weeks) compared with shControl (19.1 weeks) for line HK301 (Fig. 4D), while in HK408 genetic perturbation of E2F1 did not affect *in vivo* growth (median survival was 7 and 7.2 weeks for control and E2F1 groups, respectively). *In vivo* bioluminescent images showed tumor growth in all mice intracranially transplanted with HK408 in both control and knockdown (KD) groups (Fig. 4E). Conversely, HK301 E2F1 KD cells showed limited tumor formation *in vivo* while animals injected with HK301 control cells showed tumors in four out of five mice. We quantified the luminescence at several timepoints and found significant differences in HK301, and not in HK408, where cells treated with shE2F1 showed a marked decrease in luminescence (Fig 4F)**.** These data demonstrate that the targets uncovered by this pipeline have functional implications in patient-derived GSs.

**FIGURE 5:**
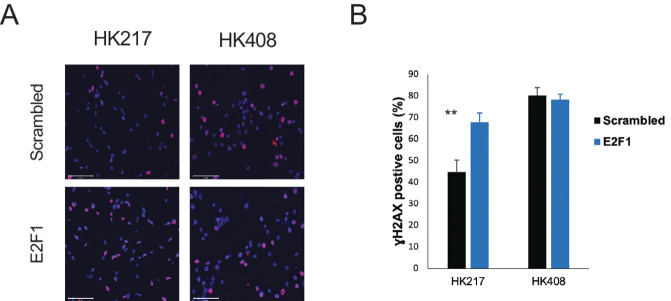
E2F1 silencing compromises DNA damage response induced after irradiation. **A,** Cells were treated with either C (control) or E (E2F1) shRNA, were subjected to irradiation (8 Gy) and fixed after 12 hours for γH2AX staining (red). Nuclei were counterstained using DAPI. **B,** Quantification for each group in A is shown. Experiment was performed at least two times. Data are represented as mean ± SEM. **, *P* < 0.01 as assessed by one-way ANOVA.

E2F1 has been demonstrated to have a role in the suppression of senescence in prostate cancer cells and proposed to be a key factor for the progression of tumors in the presence or absence of p53 or retinoblastoma (44). Similarly, we found a high enrichment in DNA repair–related genesets in the E2F1-dependent cluster (Fig. 2D). We tested this functionally by treating cells with irradiation and measuring their capacity to resolve DNA damage as measured by γH2AX staining. HK217 (E2F1-dependent) and HK408 (E2F1-independent) control and E2F1 KD cells were irradiated with a single dose of 8 Gy, and cells were stained 12 hours later (Fig. 5A). HK408 showed comparable levels of H2AX-positive cells under both conditions (control = 80%, KD = 78%, n.s.), whereas E2F1 KD significantly impacted the capacity of HK217 cells to resolve DNA damage (control = 45%, KD = 68%, *P* = 0.01; Fig. 5B). These data further confirm that the pathway-based approach we have implemented in these studies has identified a specific molecular target for a cluster of samples that has both biological significance and possible combinatorial therapeutic potential to advance treatment for pathway stratified patients with GBM.

## Discussion

In this study, rather than focusing on driver mutations themselves, our goal was to focus on their impact on gene expression and to use the latter in an unbiased manner to assay molecular pathways that will influence the biology of the tumor. We surmise that while individual mutations may influence one of several different processes, ranging from protein phosphorylation to chromatin modifications, mutations will ultimately result in altered gene expression, which then results in modified cellular function. Although our strategy does result in a reclustering of tumors, the main goal of the analyses described in this work was to present a pathway-based approach to uncover biologically relevant, potentially actionable targets derived from the heterogenous biology inherent to GBM.

Using our approach, gene signatures were established from both bulk tumor samples and patient-derived GS datasets. One of the clearest relationships, we observed was the strong correlation of signatures associated with *E2F1* activation to that of cell-cycle signatures. One potential explanation of such a finding would be that different tumors have different numbers of proliferating cells and thus differential gene expression based on their abundance. However, our findings in GSs suggest that there are more complex processes at play, as both E2F1-dependent and independent cultures were highly proliferative at the time of study, indicating that the expression differences observed represented true differences in the biology of the cells. One might assume that another closely related member of the E2F family would serve the same function as E2F1 in the nonenriched population. However, such factors would result in similar downstream effectors and therefore would not have appeared to be enriched in our studies. Similarly, the single-cell data found *E2F1* enrichment are inversely related to stem cell signatures *in vivo*, yet knockdown or genetic disruption of E2F1 in activated cultures diminished the sphere-forming capacity by limiting dilution assay. Whether this indicates that E2F1 is important for glioma stem cells (GSC) residing within tumors or whether this is because stem cell populations are artificially driven by exogenous growth factors in the GS culture system is unknown. Indeed, the two are not mutually exclusive, as E2F1 may play a role in GSCs in tumors only when they are activated to divide, a different state from what the single-cell gene signatures may be capturing.

While previous research has implicated E2F1 in the maintenance of GBM malignancy (45), our studies bring to light the fact that E2F1 may only drive proliferation in a subset of GBM cells and tumors, which we can predict using GSEA, and thus targeting E2F1 would only be relevant in these. In addition, we demonstrated the relevance of this set of genes by using fulvestrant and calcitriol that were predicted to affect only cells in the *E2F1*-dependent cluster. Fulvestrant is an estrogen receptor antagonist mainly used in breast cancer but has not been studied previously in the context of GBM. Calcitriol is an active form of vitamin D that can be used to regulate the cell cycle and induce apoptosis. It has also been associated with promoting differentiation of glioma stem-like cells and increasing susceptibility to temozolomide (46). While preliminary, these studies support our approach to identifying targetable pathways and the repurpose of FDA-approved drugs.

In addition to its role in proliferation, recent studies tie E2F1 function to other processes, including DNA repair. Our studies confirmed inhibition of E2F1 reduced the capacity to resolve irradiation-induced DNA damage in E2F1-activated GS cultures. Our analysis also identified drugs that could selectively target this pathway and be considered for development of therapeutics in subclasses of cells. Furthermore, we observed clusters that were not *E2F1* driven and that appeared to be more heavily reliant on other pathways. For example, cluster 2 in Fig. 2A showed diverse enrichment for gene lists whose main targets are more classical dysregulated pathways in GBM, such as PIK3R1 and PDGF receptor (9). This cluster also had a strong enrichment in most of its samples for an *IFNγ*- and *NFκB*-activated signature. This inflammatory and/or damage response was also observed in TCGA dataset as one of the main components for one the clusters described in our first analysis (Supplementary Fig. S2E). Finally, samples predicted to have *EGFR* activation were equally distributed in both clusters, suggesting EGFR expression levels are not particularly informative in terms of functional diversity in GBM samples.

Our use of cell signature scores rather than arbitrary values for cell type identities, allowed us to determine some of the characteristics of individual cells within tumors. This analysis confirmed that an *E2F1*-driven signature correlated with genes that were related to mitosis, but inversely correlated with putative markers of stemness. It is unclear whether this is because “true” cancer stem cells are slowly dividing, or whether other factors are involved. We were able to further our correlational analysis to include cellular states described in GBM (11) and cell types from normal brain development. The rationale to do these analyses was based on a recent report using scRNA-seq that uncovered a subset of GBM cells with outer radial-glia signatures that were able to activate an embryonic pathway to promote invasion (12). Our studies link cellular states to cell type–specific signatures and potential targets from our gene lists. For example, predicted *ICMT* activation was negatively associated with stemness and positively associated with NPC-like and OPC-like states, as well as adult OPC signatures. Similarly, IFNγ and NFκB activation were positively correlated with MES-like1 and MES-like2, as well as endothelial and mural (vasculature, pericyte) signatures. This last interaction is particularly interesting because it suggests an inflammatory environment as a driver for the expression of tumor vasculature markers. This is consistent with the capacity of glioma cells to undergo transdifferentiation into endothelial cells and pericytes to promote invasion (41–43). These analyses provide new potential avenues for the development of innovative treatments.

Recent reports have delineated the complexity of tumor cell expression signatures, and now the emphasis has been on providing a more holistic view of the cells within each tumor, such as cellular states (11), a single axis of variation between proneural and mesenchymal subtypes (47), and a recent report using a similar approach to ours that introduces another layer of complexity to GBM heterogeneity by uncovering a mitochondrial subtype with unique vulnerabilities (48). Our study adds to this trend by providing a novel approach to condense tumoral heterogeneity to critical gene lists that can be used to identify upstream regulators. In conclusion, we propose a combinatorial approach where precision medicine will be composed of sample-specific drugs that also provide specific vulnerabilities to be exploited with metabolic and/or immune-activating approaches. The integration of different aspects of a cell or sample is paramount for the development of new therapeutics.

## Supplementary Material

Table S1 Gene Lists for each PC and direction from TCGA and GS datasets

Table S2 GO terms and TF enriched in modules associated with GS clusters

Table S3 Gene signatures associate differentially to cell cycle, cellular states, and cell identities

Table S4 Normalized Enrichment Scores for each sample based on gene lists

Figure S1 Consensus clustering identifies three clusters for the TCGA dataset

Figure S2 Gliomasphere dataset analysis generates two clusters with clinical relevance

Figure S3 TCGA gene ontology analysis of clusters shows differentially enriched terms

Figure S4 Gene lists differentially correlate with cell cycle and stemness signatures

Figure S5 E2F1 targeting with multiple sgRNA validate its role in sphere formation capacity in a subset of samples

Figure S6 Validation of genelists in additional gliomasphere dataset
