# Supplementary material for: Pathway-based Approach Reveals Differential Sensitivity to E2F1 Inhibition in Glioblastoma
Source: Cancer Res Commun. 2022 Sep 23;2(9):1049–60. doi: 10.1158/2767-9764.CRC-22-0003 (PMC9536135; doi:10.1158/2767-9764.CRC-22-0003)
Supplement: Figure S1 — Consensus clustering identifies three clusters for the TCGA dataset [file crc-22-0003-s05.pdf]

Supplementary Figure 1

A Canonical Pathways

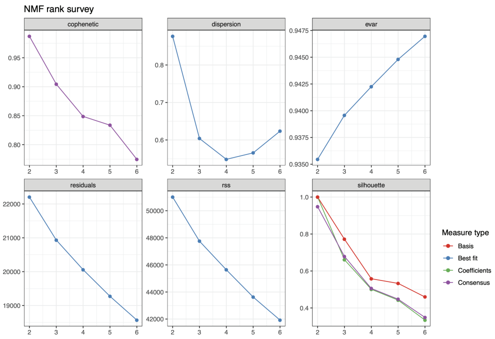

B

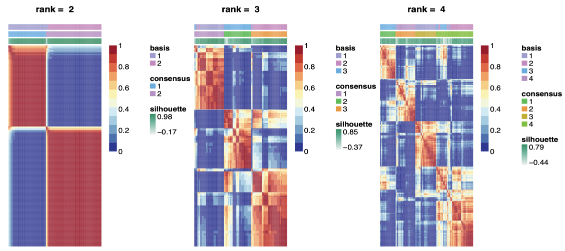

C

```
> predValidC2 <- predict(modelC2, ValidSetC2, type = "class")
> mean(predValidC2 == ValidSetC2$cluster)
[1] 0.9320988
> table(predValidC2,ValidSetC2$cluster)
```

predValidC2	1	2	3
1	**28**	2	0
2	3	**58**	4
3	2	0	**65**

D Oncogenic Pathways

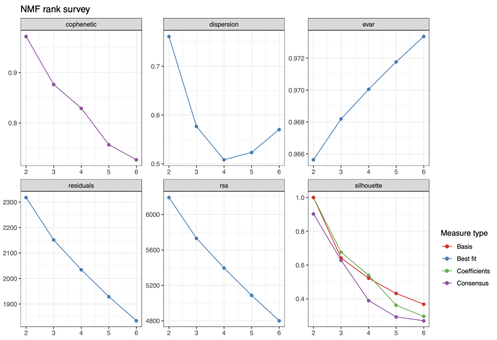

E

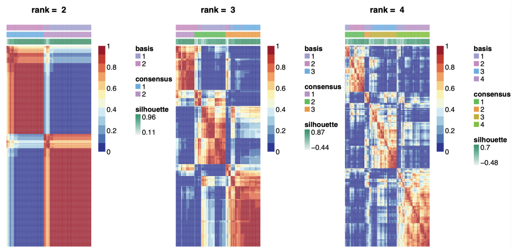

F

```
> predValidC6 <- predict(modelC6, ValidSetC6, type = "class")
> mean(predValidC6 == ValidSetC6$Cluster)
[1] 0.9753086
> table(predValidC6,ValidSetC6$Cluster)
```

predValidC6	1	2	3
1	**34**	1	0
2	1	**68**	2
3	2	0	**56**

G

Canonical Pathways

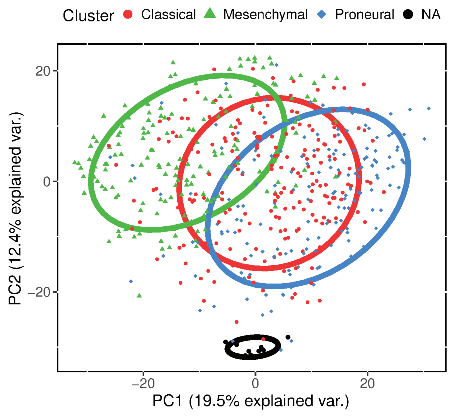

Oncogenic Pathways

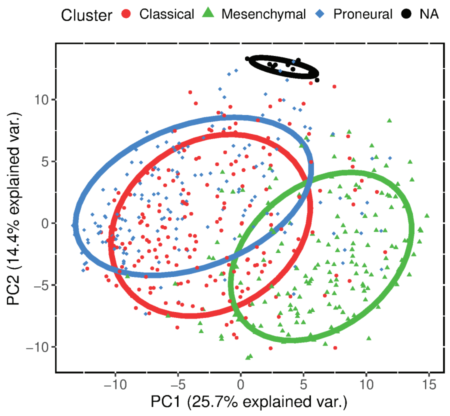

Supplementary Figure 1. Consensus clustering identifies three clusters for the TCGA dataset. (A and D) Non-negative matrix factorization (NMF) was performed with ranks ranging from 2 to 6. Various metrics are shown for canonical (A) and oncogenic (D) gene set collections. (B and E) Consensus clustering based on NMF with ranks 2 through 4, showing general structure for each rank in both canonical (B) and oncogenic (E) collections. (C and F) Random Forest classifier was used to validate the robustness of the number of clusters selected. For each analysis, canonical (C) and oncogenic (F), training sets were generated and shown are the results for the correct clustering in the validations sets. (G) Principal component analysis plots generated in Figure 1C and 1D were colored by molecular subtype in canonical (left) and oncogenic (right), respectively. Samples for which no subtype was determined are labeled as NA (black). Circle lines represent the normal distribution of the samples in each cluster.
