# Supplementary material for: Pathway-based Approach Reveals Differential Sensitivity to E2F1 Inhibition in Glioblastoma
Source: Cancer Res Commun. 2022 Sep 23;2(9):1049–60. doi: 10.1158/2767-9764.CRC-22-0003 (PMC9536135; doi:10.1158/2767-9764.CRC-22-0003)
Supplement: Figure S2 — Gliomasphere dataset analysis generates two clusters with clinical relevance [file crc-22-0003-s06.pdf]

# Supplementary Figure 2

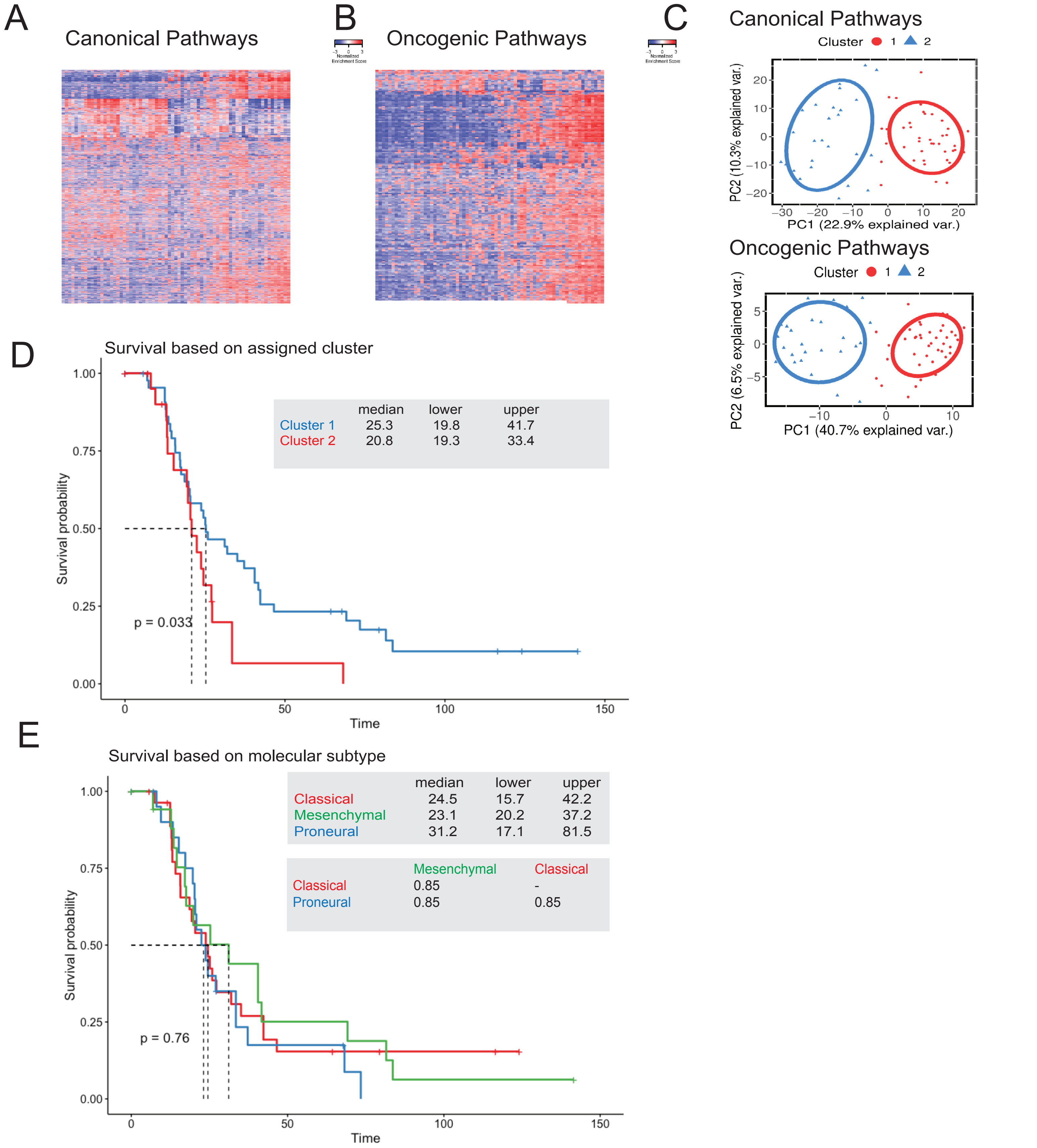


**Supplementary Figure 2.** Gliomasphere dataset analysis generates two clusters and TCGA gene ontology analysis of clusters shows differentially enriched terms. Samples from a 70-sample patient-derived gliomasphere dataset were analyzed using canonical (A) and oncogenic (B) pathways from the GSEA to generate heatmaps based on the enrichment profile of each sample (column) with respect to each gene set (row). (C) Profiles from (A) at the top and (B) at the bottom were used to generate PCA plots labeled by color and shape of each cluster. Circle lines represent the normal distribution of the samples in each cluster. Pathway-based cluster inform patient survival in gliomasphere dataset. GS samples were clustered based on the original molecular subtypes described (D) or using the assigned pathways based clusters (E), and Kaplan-Meier curves were obtained. Tables at the top describe the statistics for each subtype or cluster. Second table in A shows P-values after post-hoc analyses using Bonferroni-Hochberg correction. Dotted lines represent median survival for each curve. Time shown is in months.
