# Supplementary material for: Pathway-based Approach Reveals Differential Sensitivity to E2F1 Inhibition in Glioblastoma
Source: Cancer Res Commun. 2022 Sep 23;2(9):1049–60. doi: 10.1158/2767-9764.CRC-22-0003 (PMC9536135; doi:10.1158/2767-9764.CRC-22-0003)
Supplement: Figure S3 — TCGA gene ontology analysis of clusters shows differentially enriched terms [file crc-22-0003-s07.pdf]

Supplementary Figure 3

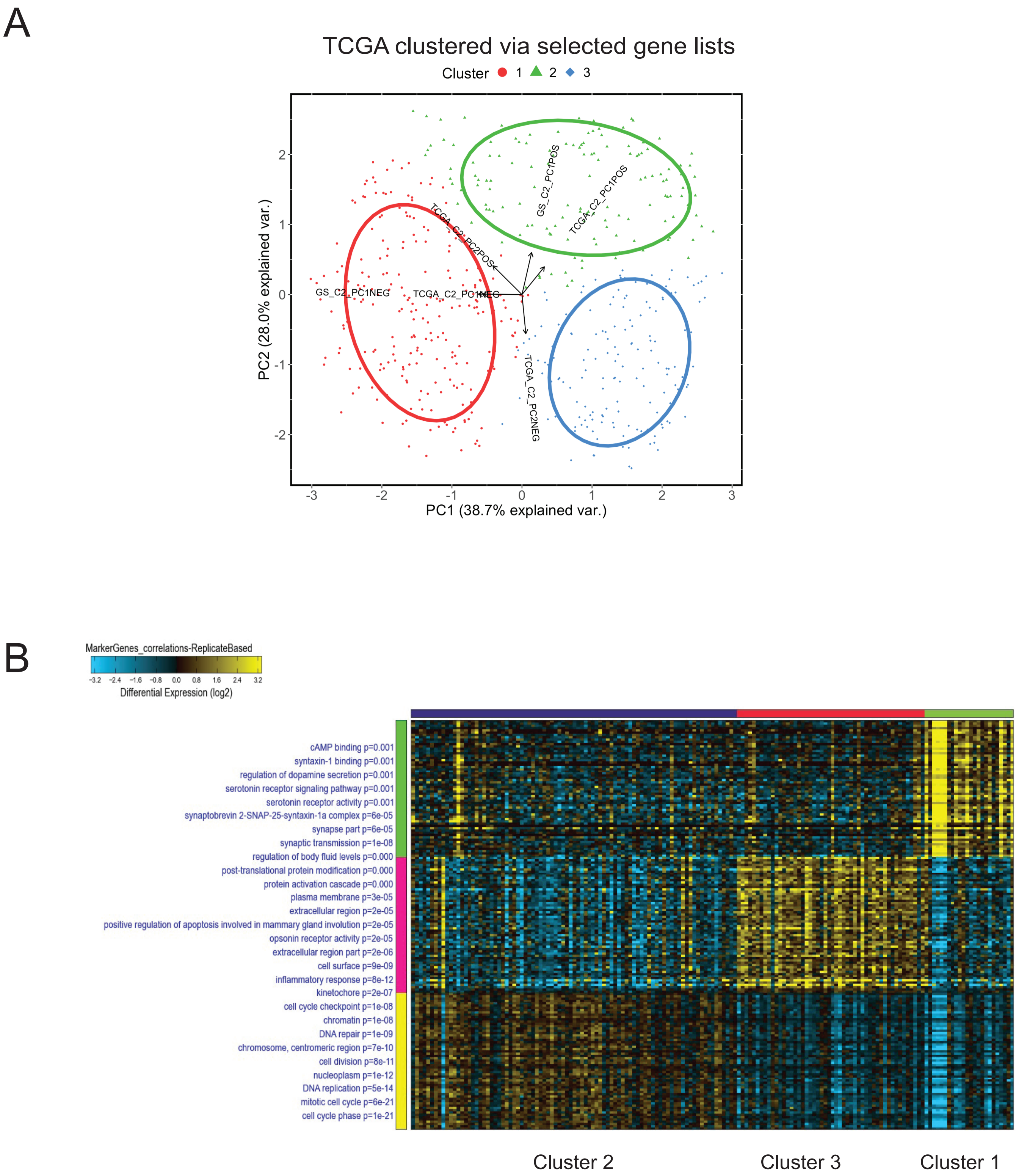

Supplementary Figure 3. (A) TCGA samples were reanalyzed utilizing the gene lists generated in Figure 2 to obtain enrichment profiles for each sample (not shown) and the corresponding shown PCA plot. The arrows show the contribution of each gene list to a particular direction in the plot. (B) The three clusters generated for the TCGA dataset were analyzed using differential expression analysis and then analyzed for the enrichment of gene ontology terms in each cluster. The plot shows the terms most highly enriched in a particular cluster compared with the other two. The expression of marker genes in each cluster is shown for every sample.
