# Supplementary material for: Pathway-based Approach Reveals Differential Sensitivity to E2F1 Inhibition in Glioblastoma
Source: Cancer Res Commun. 2022 Sep 23;2(9):1049–60. doi: 10.1158/2767-9764.CRC-22-0003 (PMC9536135; doi:10.1158/2767-9764.CRC-22-0003)
Supplement: Figure S4 — Gene lists differentially correlate with cell cycle and stemness signatures [file crc-22-0003-s08.pdf]

Supplementary Figure 4

A

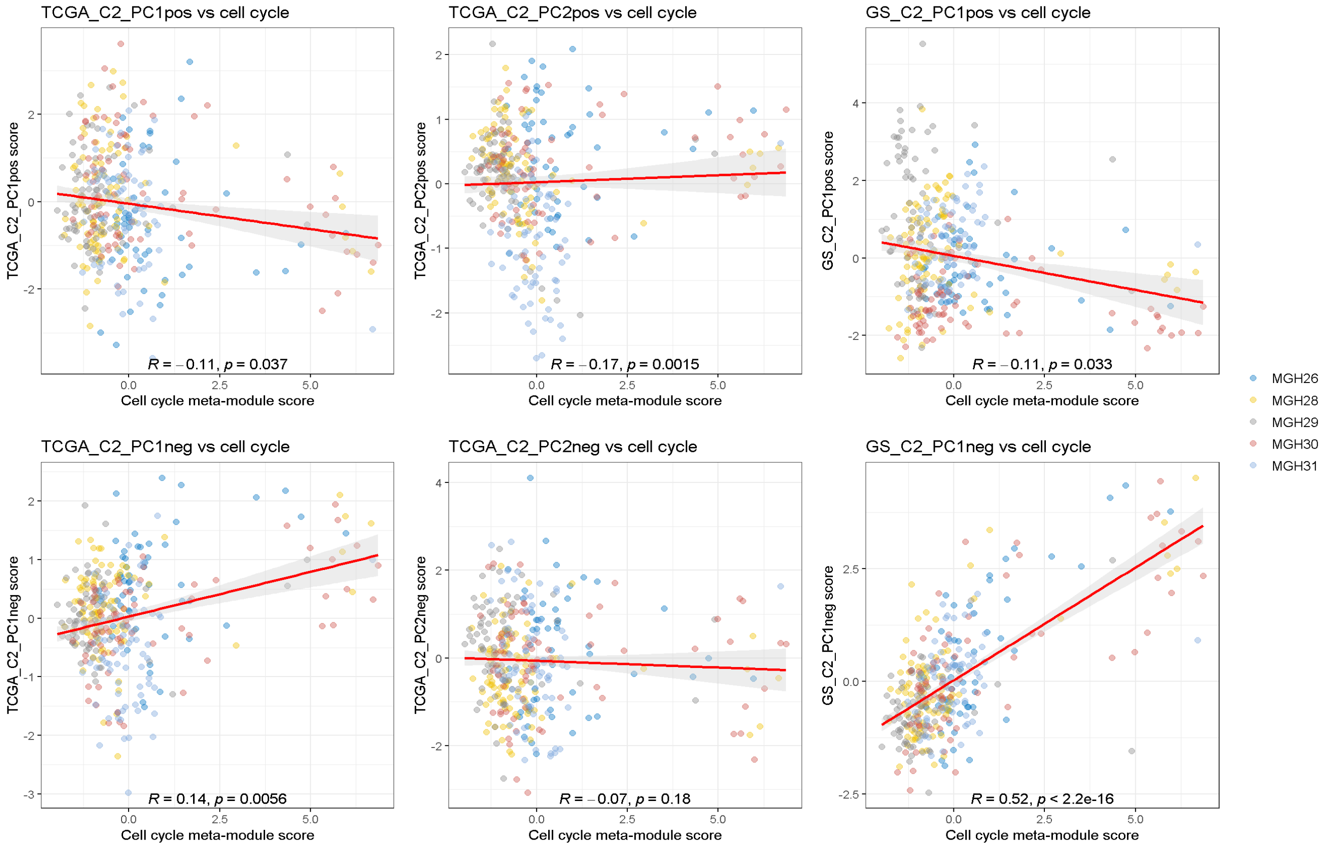

B

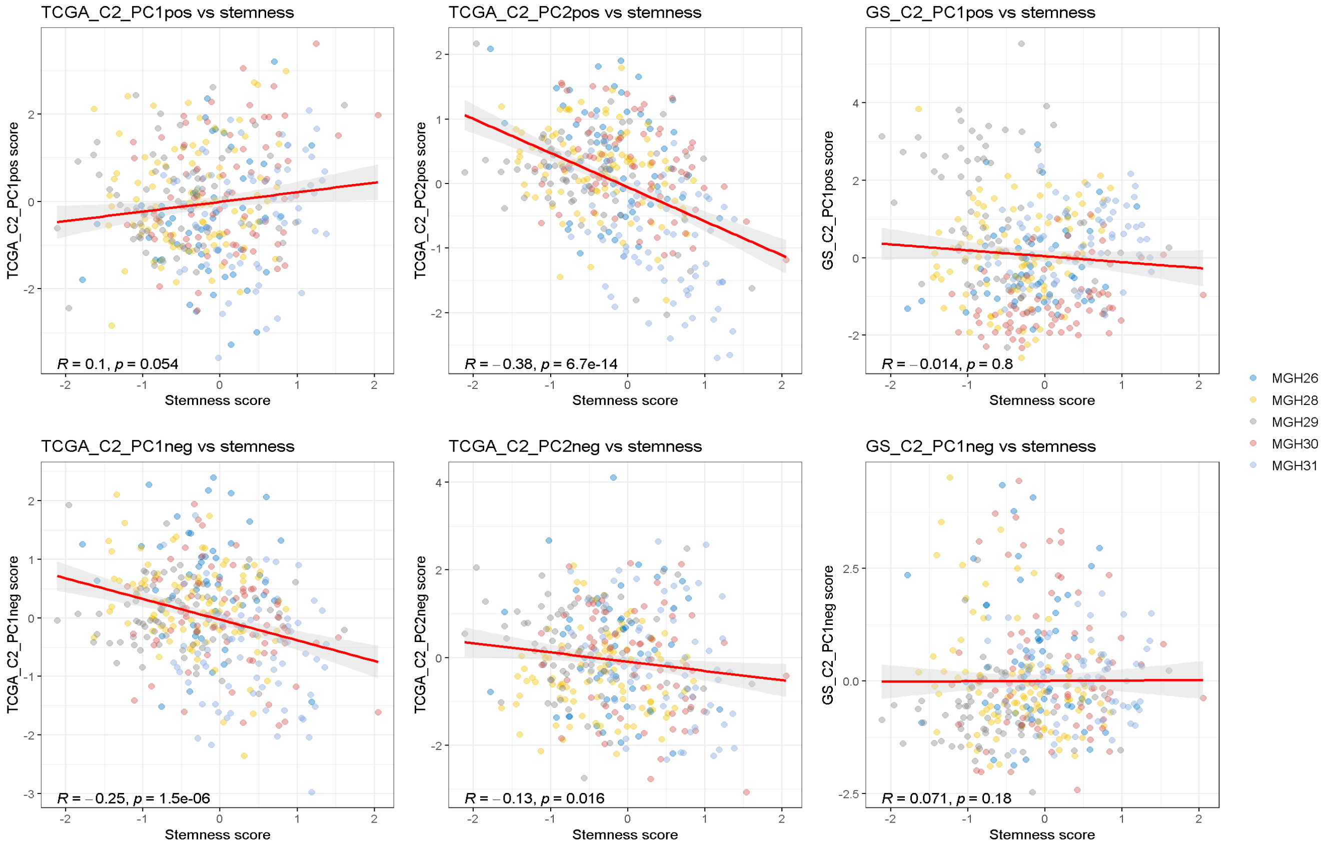

Supplementary Figure 4. E2F1 targeting with multiple sgRNA validate its role in sphere formation capacity in a subset of samples. (A and B) Samples from both clusters were treated with control (scrambled) or a E2F1 sgRNA (1-3) and plated under limiting dilution in a 96-well plate. Graphs depict the number of wells that did not form spheres after 10 days vs. the number of cells plated (a vertical line implies all wells formed spheres). Experiments were performed at least three times. Data are represented as mean +/- SEM. P-values were assessed by one-way ANOVA.
