# Supplementary material for: Pathway-based Approach Reveals Differential Sensitivity to E2F1 Inhibition in Glioblastoma
Source: Cancer Res Commun. 2022 Sep 23;2(9):1049–60. doi: 10.1158/2767-9764.CRC-22-0003 (PMC9536135; doi:10.1158/2767-9764.CRC-22-0003)
Supplement: Figure S5 — E2F1 targeting with multiple sgRNA validate its role in sphere formation capacity in a subset of samples [file crc-22-0003-s09.pdf]

Supplementary Figure 5

A

HK217 – E2F1 enriched cluster

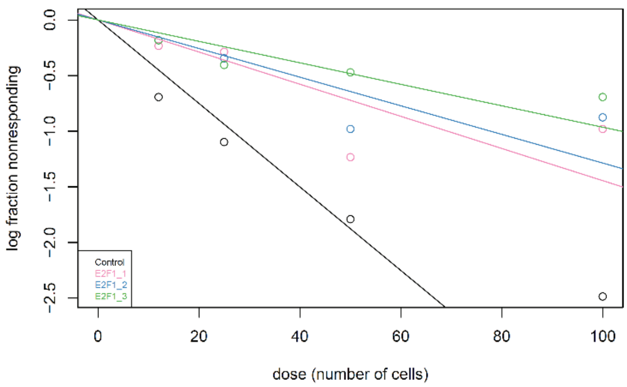

Group	Stem cell frequency	p-value vs control
Control	1/27.2	-
E2F1_1	1/69.7	5.13e-6
E2F1_2	1/78.3	4.21e-7
E2F1_3	1/104.2	4.92e-10

HK378 – E2F1 enriched cluster

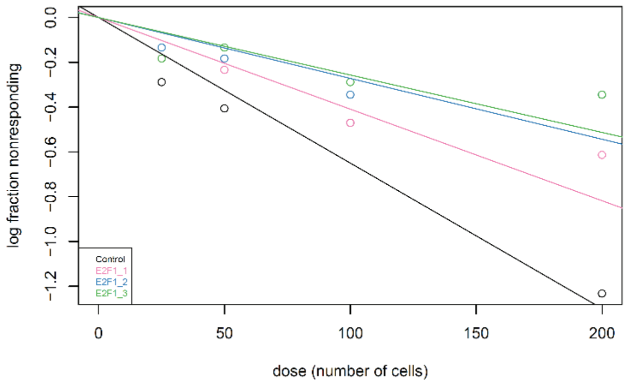

Group	Stem cell frequency	p-value vs control
Control	1/154	-
E2F1_1	1/245	0.06
E2F1_2	1/369	0.001
E2F1_3	1/391	0.0005

B

HK408 – non-E2F1 enriched cluster

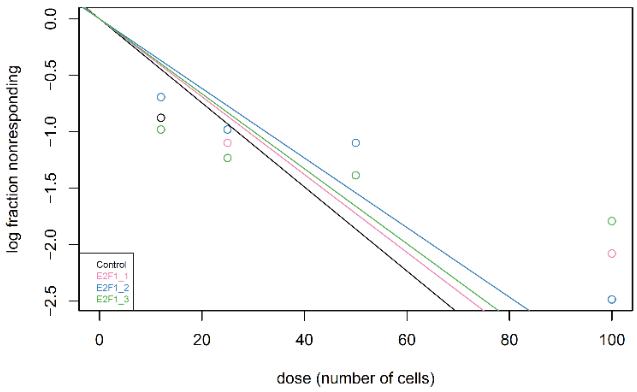

Group	Stem cell frequency	p-value vs control
Control	1/27.4	-
E2F1_1	1/29.5	NS
E2F1_2	1/33	NS
E2F1_3	1/30.6	NS

HK357 – non-E2F1 enriched cluster

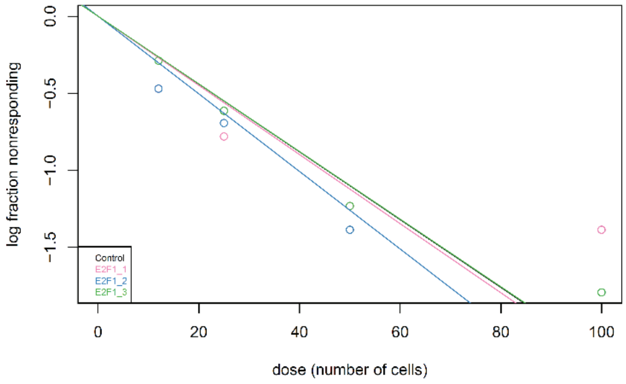

Group	Stem cell frequency	p-value vs control
Control	1/45.9	-
E2F1_1	1/45.1	NS
E2F1_2	1/40.2	NS
E2F1_3	1/46.1	NS

Supplementary Figure 5. Gene lists differentially correlate with cell cycle and stemness signatures. (A and B) Gene lists were used to obtain scores for each cell in the scRNA-seq dataset. Similarly, stemness and cell cycle score scores were generated using the signatures reported in the same paper. For each cell, we plotted the scores for cell cycle (A) or stemness (B) signatures against the scores for all the gene lists. Cells are colored by tumor of origin and p-values and correlation coefficients are shown for each plot.
