# Supplementary material for: Pathway-based Approach Reveals Differential Sensitivity to E2F1 Inhibition in Glioblastoma
Source: Cancer Res Commun. 2022 Sep 23;2(9):1049–60. doi: 10.1158/2767-9764.CRC-22-0003 (PMC9536135; doi:10.1158/2767-9764.CRC-22-0003)
Supplement: Figure S6 — Validation of genelists in additional gliomasphere dataset [file crc-22-0003-s10.pdf]

Supplementary Figure 6

A

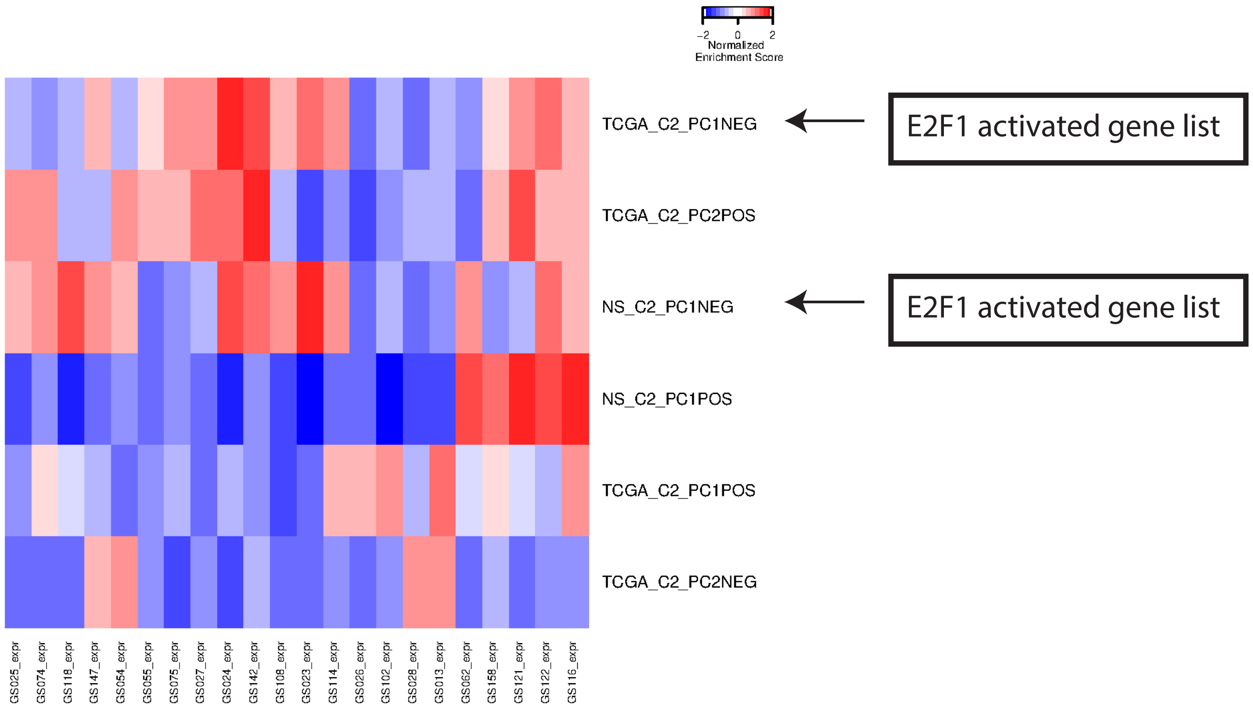

B

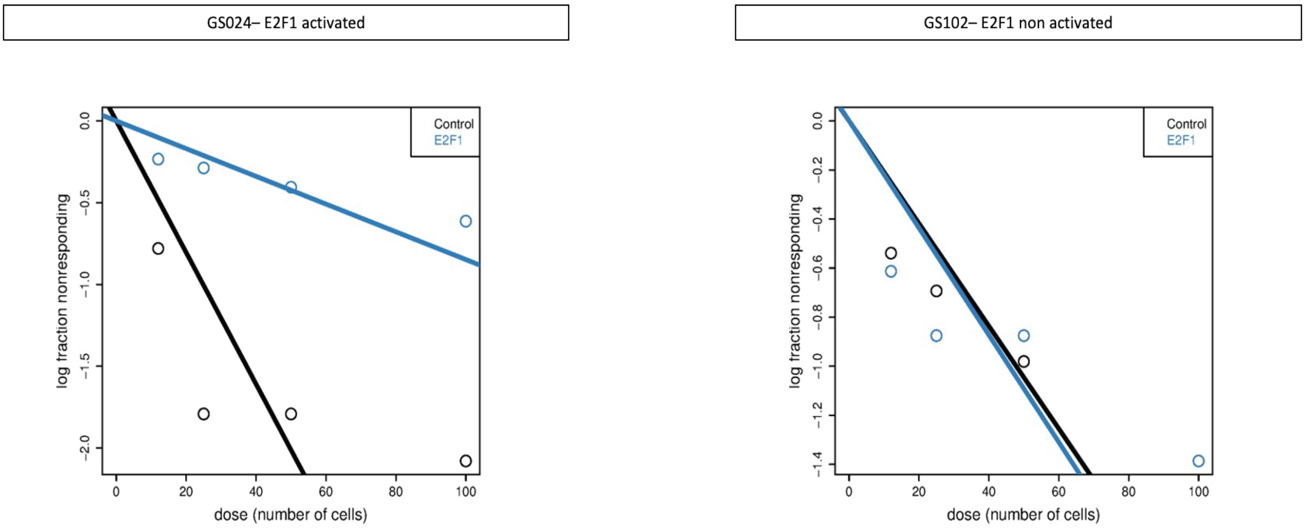

Group	Stem cell frequency	p-value vs control
Control	1/25.4	-
E2F1	1/118.5	1.1e-12

Group	Stem cell frequency	p-value vs control
Control	1/47.8	-
E2F1	1/45.8	NS

Supplementary Figure 6. (A) Normalized enrichment scores of 22 gliomasphere dataset based on previously identified gene lists. Arrows indicate gene lists where E2F1 was predicted to be activated. (B) Sphere formation analysis of GS024 (E2F1 activated) and GS102 (E2F1 non activated) after E2F1 knockdown compared with control knockdown.
